# Aseptic Meningitis in Oral Medicine: Exploring the Key Elements for a Challenging Diagnosis: A Review of the Literature and Two Case Reports

**DOI:** 10.3390/ijerph19073919

**Published:** 2022-03-25

**Authors:** Stefania Leuci, Noemi Coppola, Tiziana Cantile, Elena Calabria, Laurenta Lelia Mihai, Michele Davide Mignogna

**Affiliations:** 1 Oral Medicine Unit, Department of Neurosciences, Reproductive and Oral Sciences, “Federico II” University of Naples, 80131 Naples, Italy; stefania.leuci@unina.it (S.L.); noemi.coppola@unina.it (N.C.); calabriaelena92@gmail.com (E.C.); mignogna@unina.it (M.D.M.); 2 Facultatea de Medicina Dentară, Universitatea Titu Maiorescu, 040051 Bucharest, Romania; lelia_mihai2000@yahoo.com

**Keywords:** aseptic meningitis, oral medicine, Behçet’s disease, Sjögren syndrome, intravenous immunoglobulin, herpes simplex virus, hand, foot, and mouth disease, drug-induced aseptic meningitis case, Behçet’s disease related aseptic meningitis case

## Abstract

Aseptic meningitis (AM) is a potentially severe and life-threatening disease characterized by meningeal inflammation, usually with mononuclear pleocytosis. It represents a challenging and controversial issue in medicine for multiple etiologies, classification, and difficult diagnosis in the face of nonspecific sets of signs and symptoms. In the area of interest of oral medicine, in specific clusters of patients, even if rare, the occurrence of aseptic meningitis can pose a diagnostic and management dilemma in the following potential etiologies: (i) systemic diseases with oral and meningeal involvement, which include Behçet’s disease and Sjögren syndrome; (ii) drug-induced aseptic meningitis; (iii) aseptic viral meningitis, mostly related to herpes simplex virus infection and hand, foot, and mouth disease, caused by enteroviruses. In this review, clinical manifestations, diagnostic methodologies, incidence, treatment, and prognosis for each of these clinical entities are provided. Furthermore, two illustrative case reports are described: a patient suffering from recurrent oral ulcers, in which a sudden onset of AM allows us to diagnose Neuro Behçet’s disease, and a patient affected by pemphigus vulgaris, manifesting a drug-induced AM. Exploring this complex clinical entity scenario, it is clear that an oral medicine specialist has a place on any multidisciplinary team in making such a challenging diagnosis.

## 1. Introduction

Aseptic meningitis (AM) is defined as an illness characterized by inflammation of the meninges, usually with cerebrospinal fluid (CSF) pleocytosis (CSF white blood cell (WBC) >5 cells/mm^3^) in the absence of a positive Gram stain and culture, caused by different etiologies [1].

The annual incidence of AM is 7.6 per 100,000 adults, with no specific association with sex or age [2]. AM is usually mild with a self-limited course, but some cases can be severe and at risk of death [3]. In particular, infants usually present the most severe symptoms and immunocompromised patients are at a higher risk of negative outcomes [4,5].

Etiologies of AM can be categorized as infective and non-infective.Among the former, viral meningitis represents most of the cases [4], and it is commonly called “aseptic” meningitis, even if the term is inaccurate, in clinical practice [6].

The most common causes of AM are viral infections, where enteroviruses represent the vast majority of cases. These are small, non-enveloped RNA viruses, belonging to the picornavirus family, comprising over 70 different serotypes. Coxsackieviruses and echoviruses are responsible for approximately half of all cases of AM [3,5,7] with an incidence that usually increases in the summer and early fall. The transmission of the enteroviruses occurs through the fecal–oral route, although inhalation of infected droplets and hand-to-mouth contact have also been described. The incubation period is 3–6 days [3,5].

Other viral causes include: herpes simplex viruses, both labialis (HSV-1) and genital (HSV-2), West Nile virus, varicella zoster virus (VZV), cytomegalovirus (CMV), human immunodeficiency virus (HIV), arboviruses, mumps, Epstein–Barr virus (EBV), adenovirus, influenza virus, lymphocytic choriomeningitis virus [4,5].

Non infective AM can be caused by systemic diseases with meningeal involvement, malignancies and drugs [8] (Table 1).

The general clinical findings include: fever, headache, photophobia, phonophobia, neck stiffness, nausea, vomiting, arthralgia, myalgia, rash (e.g., VZV and enterovirus infections), abdominal pain, irritability, sore throat, altered mental status [7]. Clinical presentation may be slightly different according to the patient’s age. In fact, older patients are more likely to have altered mental status and focal neurologic deficits. Otherwise, in young children, vague symptoms, such as irritability, lethargy, or poor feeding can be present, as well [2].

Nonspecific signs that may suggest meningeal irritation comprise positive Kernig sign (severe stiffness of the hamstrings causes an inability to straighten the leg when the hip is flexed to 90 degrees), positive Brudzinski sign (severe neck stiffness causes a patient’s hips and knees to flex when the neck is flexed), and jolt accentuation of headache (worsening of headache by horizontal rotation of the head two to three times per second) [2]. An in-depth evaluation, including personal and familiar medical anamnesis, geographical setting, season of the year, patients contacts, history of recent trips, and recent drugs assumption, could be useful for a plausible diagnosis [3,4].

When meningitis is supposed, a lumbar puncture should be performed as soon as possible. Head computed tomography (CT) should be carried out before lumbar puncture when elevated intracranial pressure is suspected to avoid the risk of cerebral herniation. However, head CT is not necessary when all these findings are absent: age equal to 60 or greater than 60, previous history of CNS disease, immunocompromised or altered mental status, seizure during the previous week, and neurological deficits [4,5]. CSF laboratory tests allow the obtainment of cell count, glucose, gram stain, protein, culture, bacterial PCR (e.g., *for N. meningitides, S. pneumonia, H. influenza*), and viral PCR (e.g., *for enterovirus, HSV-1, HSV-2, VZV, CMV, EBV, arbovirus*) [4].

CSF characteristic findings in patients with AM are clear appearance, normal or elevated opening pressure, usually normal glucose, normal to moderately increased proteins (>50 mg/dL), and cell count of 10–1000 cells per microliter (early: neutrophils; late: lymphocytes) [4].

Furthermore, serum C-reactive protein and CSF lactate levels may also be elevated in patients with viral infections [5]. Instead, serum procalcitonin is highly sensitive and specific for bacterial meningitis [2]. However, despite the improvement in diagnostic procedures, the cause of AM can be clearly recognized in only 30% to 65% of cases [5].

Owing to the similarity between the clinical and laboratory findings for bacterial meningitis and AM, it may be difficult to make a proper differential diagnosis [5].

First, it should be considered that, although presentation can be similar, patients with bacterial meningitis may appear more critically ill. Furthermore, on CSF analysis, bacterial meningitis generally presents with elevated opening pressure, a very high and predominantly neutrophilic pleocytosis, low glucose level, and high protein level. CSF fluid can appear clear, cloudy, or purulent [2].

In addition to bacterial meningitis, other diseases, such as intracranial hemorrhage (in particular subarachnoid hemorrhage), malignancies, other types of headaches (migraine), and inflammation of brain structures (brain abscess, epidural abscess) should also be taken into account for alternative differential diagnosis [4].

AM is usually treated with no specific pharmacologic therapies; only supportive measures, including analgesics, anti-nausea drugs, and intravenous fluids, are required [3,5]. For the drug-induced variant, the responsible molecule must be promptly interrupted, while when an enteroviral infection is suspected, patients should be adequately instructed for body and food hygiene. When HSV and/or VZV is suspected, acyclovir should be administered [3,4]. Finally, if bacterial etiology is suspected, empiric antibiotics, with a broad antimicrobial spectrum, should be started while waiting for the results of bacterial culture. In addition, dexamethasone should be administered [2,5].

The prognosis of the disease is usually benign, with low rates of morbidity and mortality. Healing usually occurs within 5 to 14 days. However, tiredness and dizziness may persist for months [5].

Complications can be represented by seizures or status epilepticus. Furthermore, cases of encephalitis have been reported with viral meningitis. Sequelae of mumps meningoencephalitis can be represented by deafness and aqueductal stenosis, leading to hydrocephalus [9].

In the field of interest of oral medicine, the occurrence of AM cases should be explored.

## 2. Materials and Methods

MEDLINE/PubMed and Web of Science literature searches were performed, using the following keywords in different combinations: “aseptic meningitis”; “oral medicine”; “oral”; “oral cavity”; “oral diseases”; “oral syndromes”; “viral infection”.

Clinical manifestations, diagnostic methodologies, incidence, treatment, and prognosis for each clinical entity analyzed are provided. Furthermore, two illustrative AM case reports involving oral medicine patients—a woman affected by Behçet’s Disease (BD) and a woman undergoing intravenous immunoglobulin (IVIg) therapy due to pemphigus vulgaris (PV)—are described.

## 3. Results

Based on the results obtained, the causes of aseptic meningitis in oral medicine can be classified as follows: (i) immune-mediated systemic diseases with meningeal involvement, which include Behçet’s disease (BD) and Sjögren syndrome (SS); (ii) drug-induced aseptic AM; (iii) aseptic viral meningitis, mostly related to HSV infection and hand foot, and mouth disease (HFMD), caused by enteroviruses.

### 3.1. Behçet’s Disease

(BD) is a chronic multisystemic inflammatory disease, characterized by recurrent oral and genital ulcers, with a frequency of 97–99% and 85%, respectively [10], ocular, skin and vascular lesions, arthritis, and neurological manifestations [11]. The highest prevalence is in the areas extending from Asia through the Middle East into European countries around the Mediterranean, and the susceptibility is associated with HLA-B51 allele [12,13]. Clinical manifestations appear between the second and the fourth decade of life; males usually show a more severe symptomatology and a more frequent neurological impairment [12].

Diagnostic criteria include the presence of recurrent oral involvement in addition to two of the following features: eye disease (uveitis or retinal vasculitis), skin disease (erythema nodosum, pseudo folliculitis, papulopustular lesions, acneiform nodules), recurrent genital ulcers, positive pathergy test.

Oral involvement is characterized by recurrent episodes of aphthous ulcers, which are the initial signs in 80% of patients, preceding the other manifestations by about 7–8 years. Oral ulcerations are painful with erythema around the lesions. Pseudomembrane may be found. The non-keratinized portions of the oral mucosa, such as the soft palate, lips, and buccal mucosa, are the most frequent localizations. On the basis of the size and the arrangement, in BD minor and major ulcers are the most common type, whereas herpetiform types are rarely found. Oral ulcers appear about 10 times a year, with a healing time ranging from 10 days to 4 weeks [12]. On histopathology, BD is classified as an occlusive vasculitis, involving both arterioles and veins [14].

Central nervous system (CNS) involvement (known as Neuro-Behçet’s disease (NBD) occurs in 5–30% of patients and constitutes a negative prognostic factor [10].

It can present with parenchymal involvement, in which meningoencephalitis occurs, or with vascular involvement, frequently manifesting with cerebral venous thrombosis, stroke, and aneurysms [10,15].

The incidence of AM in NBD cases is 3.7–7.5%, usually presenting with the involvement of brain stem (Table 2).

CSF analysis frequently shows a predominance of neutrophils and increased proteins. Characteristic findings are fever, generalized headache, neck stiffness, and signs of meningeal irritation [15].

Furthermore, cases of isolated AM without any other systemic BD manifestations, although quite rare, are also described [23].

NBD must always be listed as a differential diagnosis of acute meningeal syndrome unless an infectious disease is recognized, because prompt diagnosis and treatment are fundamental for reducing morbidity [14].

In particular, a correct diagnosis is important for patients without known systemic BD, since NBD can present as the first manifestation of BD [12].

The differential diagnosis for NBD includes infectious, autoimmune, inflammatory, and neoplastic diseases. Rheumatologic and inflammatory disorders to take into consideration are systemic lupus erythematosus and SS and neuro-sarcoidosis. Furthermore, tuberculosis, neuro-syphilis, and viral and fungal infections must be excluded, together with carcinomatosis and lymphomatosis [12].

BD-related AM, when managed with steroid therapy, usually shows a favorable prognosis [19].

### 3.2. Sjögren Syndrome

SS is an autoimmune inflammatory disorder, characterized by mononuclear lymphocytic infiltration of salivary and lacrimal glands, leading to a secondary chronic impairment that results in ocular and oral dryness, the so called “sicca syndrome” [34].

In addition to marked hyposcialia, oral manifestations of SS include burning, taste disturbances, and discomfort during swallowing and during prolonged speaking. Furthermore, Candida albicans infections are 10 times more frequent in patients with SS than in the general population. Bilateral, chronic, or episodic, parotid swelling occurs in approximately 30% of patients. This clinical manifestation requires screening for non-Hodgkin lymphoma B cell lineage. Furthermore, with oral involvement, the more common dental findings are a higher incidence of caries than in the general population and early tooth loss [70].

SS is described as an isolated clinical condition (primary SS) or occurring in association with other autoimmune disorders (secondary SS), such as rheumatoid arthritis and systemic lupus erythematosus [26].

It can be considered the second most common systemic autoimmune disease after rheumatoid arthritis, with a prevalence in the adult population of 0.1–0.6% and a female to male ratio equal to 9:1.

The disease generally appears between the fourth and the fifth decade of life, but it may also occur in childhood and in old age [34].

As for other autoimmune disorders, although the exact cause is unknown, it is believed to involve a combination of genetics and an environmental trigger, such as exposure to a virus (in particular EBV) [70].

When SS is suspected, a classification/diagnostic criteria set needs to be applied in line with the two major reports from the American–European Consensus Group (AECG) and the Sjögren’s International Collaborative Clinical Alliance (SICCA-ACR), which have proven useful in research and clinical practice [71,72].

One-third of patients with primary SS can show extra-glandular manifestations, such as arthritis, Raynaud’s phenomenon, lymphadenopathy, vasculitis, pulmonary, renal, and neurological diseases, and malignant lymphoma [26].

The incidence of neurologic manifestations is highly variable and ranges between 8.5% and 70% [73]. This discrepancy can be due to heterogeneity of neurologic manifestations, various classification criteria used, or unclear inclusion or exclusion criteria [74].

CNS manifestations are detected in 14–19% of cases, often before the diagnosis of primary SS, and occur as recurrent episodes, spaced out by long periods without clinical signs, leading to a gradual neurologic deficit [28,74]. Furthermore, CNS involvement can result in severe disability despite SS treatment in 40% of the cases [73].

The clinical spectrum of CNS manifestations includes focal (sensorial and motor deficits, brain stem, cerebellar lesions, seizure, migraine, etc.) or non-focal (encephalomyelitis, AM, neuropsychiatric dysfunctions) or spinal cord (myelopathy, transverse myelitis, motor neuron disease etc.) findings, or multiple sclerosis-like illness and optic neuritis [74].

Three different pathogenic mechanisms are supposed to be involved in CNS diseases: direct infiltration of the CNS by mononuclear cells, vascular injury due to the presence of antineuronal antibodies and anti-Ro antibodies, and ischemia secondary to small vessel vasculitis [31].

AM constitutes about 20% of the CNS manifestations of primary SS, presenting as an acute, sometimes recurrent, process with variable outcomes [26] (Table 2).

The acute presentation is characterized by several meningitis features, including headache, neck stiffness, and rarely, focal neurological findings and seizure [27].

The cerebrospinal fluid (CSF) can show variable pleocytosis, consisting mainly of polymorphonuclear leukocytes at the beginning, followed by mononuclear cells. Oligoclonal bands of immunoglobulins in the CSF have also been described [26,27].

The meningeal inflammatory pattern can be nonspecific, represented by a lymphocytic infiltration or in association with vasculitis of the CNS [26].

CT and magnetic resonance imaging (MRI) may be normal or show non-disease-specific findings, such as diffuse leptomeningeal and subarachnoid enhancement [27].

Treatment usually consists of corticosteroid administration, and most cases can be resolved with this therapy alone. Cyclophosphamide, azathioprine, cyclosporine, methotrexate, chlorambucil, and tacrolimus, alone or in association with corticosteroids, may also be useful. Recently, anti-CD20 monoclonal antibody (rituximab) has been reported to successfully treat CNS manifestation of SS [27].

SS should be included in the differential diagnosis of recurrent AM of unknown etiology to improve prognosis and prevent complications [73].

### 3.3. Drug-Induced AM

Drug-induced aseptic meningitis (DIAM) represents a challenging and difficult diagnosis where infections must be firstly excluded, and diagnosis is based on exclusion criteria, depending on the establishment of a causal relationship with the drug concerned [3]. In selected cases and under appropriate supervision, rechallenging the patients with the potentially responsible molecule is needed to confirm the diagnosis. Evaluation of the timing of administration, early onset after a systemic therapy, and a subsequent fast resolution after withdrawal of the suspected drug are the main elements for making a proper diagnosis [3,4].

DIAM could be explained by direct meningeal irritation due to intrathecal medication and type III and IV hypersensitivity reactions [3]. The clinical features are with the same as for infective and non-infective AM, and CSF analysis usually shows pleocytosis and polymorphonuclear predominance. Moreover, the protein levels in CSF are usually elevated, whereas the concomitant glucose level in CSF remains normal. DIAM is managed with discontinuation of the causative drug.

Drugs that can cause aseptic meningitis include the following: antimicrobials (trimethoprim-sulfamethoxazole, ciprofloxacin, cephalexin, metronidazole, amoxicillin, penicillin, isoniazid); nonsteroidal anti-inflammatory drugs (NSAIDs); intravenous immunoglobulins (IVIGs); vaccines; intrathecal medications (methotrexate, cytosine arabinoside); monoclonal antibodies (Muromonab-CD3); ranitidine; carbamazepine; lamotrigine; azathioprine; allopurinol; sulfasalazine [3,4].

Among these, drugs commonly used in dentistry and mostly in oral medicine are antimicrobials, NSAIDS, and in selected cases, intravenous immunoglobulins (IVIgs), azathioprine and sulfasalazine. Up to now, current published literature shows only one paper related to a case of mucocutaneous pemphigus vulgaris treated with IVIgs who developed DIAM [46] (Table 2).

#### IVIg Therapy

Human immunoglobulins (Igs) are used for a broad range of diseases, including replacement therapy in primary and secondary immunodeficiencies, prevention and treatment of certain infections, and as an immunomodulatory agent for autoimmune and inflammatory disorders [75].

Immunoglobulins are therapeutic preparations comprising pooled blood from thousands of healthy donors. IgG is the main component, but small amounts of other Ig classes (most IgA) and varying trace amounts of maltose, sucrose, albumin, and salts are present [48,76].

Over the last two decades, IVIg therapy has been successfully applied in a variety of autoimmune diseases, and, in our field of interest, in autoimmune mucocutaneous blistering diseases (AMBDs) as a second- or third-line treatment [77].

Ig can be given intravenously, intramuscularly, or by subcutaneous infusions. The intravenous route is generally well tolerated, avoids painful intramuscular injections, and allows larger doses, resulting in rapid achievement of therapeutic serum IgG levels and in significant improvements in patients’ conditions [75].

Immunomodulatory and anti-inflammatory conditions may require high doses (typically 1 to 2 g/kg per dose, repeated at regular intervals), but effects usually continue beyond the circulating IVIg half-life [75,78].

The IVIg immunomodulatory and anti-inflammatory mechanisms are still not completely understood and may involve several biological pathways. The most accredited effects are mediated by the antigen-binging fragment (Fab) of IgG and by the crystallizable fragment (Fc). The Fab-dependent effects can be summarized as follows: reduction in the production or neutralization of proinflammatory cytokines; suppression or neutralization of autoantibodies; down-regulation of adhesion molecules and chemokines; neutralization of superantigens and of activated complement components; restoration of idiotypic–anti-idiotypic networks; modulation of maturation and function of dendritic cells. Fc-mediated effects are the blocking of Fc receptors and immunomodulation by sialylated IgG [78,79].

Although IVIg therapy is well tolerated, various adverse effects have been reported, occurring in 10–30% of IVIg infusions. Most adverse effects are common and benign, but some rare side effects are more serious, including AM, renal failure, thrombosis, and hemolytic anemia [76,78].

Adverse reactions to Igs can be local or systemic. Local adverse reactions include pain, bruising, swelling, and erythema at the needle site. Systemic adverse reactions can be immediate, manifesting during or within 6 h from the infusion; delayed, manifesting 6 h–1 week after the infusion; or late, manifesting weeks and months after the infusion [75].

Immediate systemic reactions, such as headache, nausea, malaise, myalgia, arthralgia, fever, chills, chest discomfort, skin reaction, fatigue, dyspnea, back pain, vomiting, diarrhea, changes in blood pressure, and tachycardia, are usually mild and readily treatable [80].

Delayed adverse effects can be severe, and include thrombotic events, neurological disorders, renal impairment, hematologic disorders, electrolyte disturbance, and transfusion-related infection. Neurological disorders associated with immunoglobulin treatment include headache, AM, posterior reversible encephalopathy syndrome (PRES), seizure, and abducens nerve palsy [76].

Late reactions are uncommon, but often severe, and include lung disease, enteritis, dermatologic disorders, and infectious diseases [75].

Among these adverse reactions, if AM occurs it usually manifests within a few hours to a few days after IVIg administration [78]. The mechanism by which IVIg therapy can cause AM is not completely understood, and it is supposed to be more complex than direct meningeal irritation. Suggested mechanisms include cerebral vasospasm, neutrophil activation, and hypersensitivity reaction [47].

IVIg-induced AM has the same symptoms as other forms of AM [76].

Cerebral spinal fluid analysis usually shows pleocytosis, with a polymorphonuclear predominance, elevated proteins level, normal glucose concentration, and negative culture results. Neuroimaging is usually not useful [48]. The onset of AM has been related to a previous history of migraine headaches and to high-dosage administration over a short period [76]. The symptoms of meningitis are self-limiting and the majority of patients usually recover within 5 days of the onset of symptoms [80]. Treatment is primarily supportive and may include fluids, analgesics, anti-emetics and anti-migraine therapy. Corticosteroids are not considered beneficial [44,80].

If IVIg therapy must be continued, infusions should be administered in smaller increments, with slower rates and with a different IVIg brand [75].

The picture of IVIg-induced AM may be difficult to differentiate from other forms of meningitis, but the temporal relation between IVIg administration and onset of the meningeal symptoms can be helpful to make a prompt diagnosis [48].

### 3.4. Herpes Simplex Virus

Herpes simplex virus type 1 (HSV-1) and type 2 (HSV-2) are members of the large family of herpesviruses.

They are characterized by a short reproductive cycle, rapid destruction of the host cell, and the ability to establish latency within sensory ganglia [81].

They both occur worldwide in developed and developing countries without seasonal variation, with a seroprevalence of HSV-1 and HSV-2 equal to 67% and 11.3%, respectively [82,83].

Primary HSV infection is defined as infection with one HSV subtype in patients with no existing antibodies to HSV-1 or HSV-2. Non-primary HSV initial infection is defined as infection with one HSV subtype in patients who already have antibodies to the other HSV type. Recurrent HSV infection is defined as infection in a patient who is seropositive for either and develops a recurrence of this infection [84].

HSV-1 and HSV-2 are closely related, with nearly 70% genomic homology. Despite this, there are significant differences in their clinical manifestations. In fact, though not exclusive of each other, HSV-1 is more commonly associated with herpes labialis, whereas HSV-2 is a more frequent cause of genital herpes [56].

Herpes viruses can be responsible for significant neurological morbidity. In fact, HSVs enter the brain mainly via the peripheral and cranial nerves. Once the viral agent enters the CNS, increased levels of chemo-attractants, neutrophils, CD8 T cells, and monocytes are detected, indicating the induction of an immune response [85].

In particular, HSV-1 is the most common cause of viral encephalitis. Less frequently, HSV-1 can cause meningoencephalitis, and rarely, AM, usually after a recent episode of primary HSV-1 infection [52] (Table 2). By contrast, HSV-2 is more frequently associated with AM in adults and CNS infection in neonates [52].

Viral AM patients present with fever, severe headache, malaise, nuchal rigidity, photophobia, and lethargy, but no cerebral dysfunction should be present [81]. Encephalitis patients may show motor or sensory deficits, altered mental status, disturbed consciousness, and seizures. Since a clear distinction can be challenging, classification is usually based on the predominant involvement of meninges (meningitis), brain parenchyma (encephalitis), or both (meningoencephalitis) [86].

Viral infection among cases of AM has been studied in many areas in the world, and several rates of infection have been reported [54].

CSF of patients with HSV-1 encephalitis presents mononuclear predominance of cells, variably elevated protein, normal glucose, and red blood cells (RBCs), because of the hemorrhagic/necrotizing nature of the infection. On the other hand, on CSF analysis, HSV-1 AM usually shows higher white blood cell counts and increased proteins, with no RBCs [52].

While HSV-1 AM is more benign and self-limited and usually resolves without residual sequelae, HSV-1 encephalitis often results in permanent neurologic sequelae and carries a mortality of 20–50% when treated [52,81].

Diagnosis is established through clinical history and PCR confirmation of viral infection [87]. Treatment with antiviral therapy may be beneficial in shortening the duration of the illness, decreasing severity of symptoms, or preventing recurrences, although this is not yet established by controlled studies [81].

Furthermore, scientific literature reports several cases of benign recurrent AM, known as Mollaret’s meningitis, first described by French neurologist Pierre Mollaret in 1944. It is mostly observed in young adults, with a female predominance [88]. It is characterized by 3–10 episodes of fever and signs of meningeal irritation, lasting between 2 and 5 days, with spontaneous recovery, followed by periods during which signs and symptoms are absent and the CSF is normal [88].

The rate of recurrence can be variable, with episodes ranging in frequency from every few weeks to intervals of many years [89]. HSV-2 is the most commonly isolated pathogen in this condition, followed by HSV-1 [88].

Its clinical presentation is similar to other forms of meningitis. About one-half of patients have transient neurologic manifestations, with seizures, hallucinations, diplopia, and cranial nerve palsies, but no permanent impairment [89].

CSF analysis can show clear CSF, pleocytosis, lymphocytosis, unusual large lymphocytes or mononuclear cells, and endothelial cells (Mollaret cells) [89]. The protein concentration can be mildly elevated, and the glucose concentration is usually normal. CSF viral culture is usually negative [88].

Mollaret cells are large monocytic cells with nuclei shaped like a bean, footprint, or cloverleaf, and deep nuclear clefts. Large, degenerated cells, termed “ghost cells”, can be also present [89]. Mollaret cells are highly fragile and tend to lyse, explaining their rapid disappearance from the CSF after 24 h from the onset of the symptoms [90].

Similar monocytic cells have also been described in other pathologies, such as sarcoidosis, BD, VZV, and SS, decreasing the specificity for Mollaret’s meningitis [90].

Mollaret’s meningitis is a self-limiting and benign disease and should be treated with supportive measures only [89].

### 3.5. Hand, Foot, and Mouth Disease

Hand, foot, and mouth disease (HFMD) is a commonly occurring infectious disorder in children caused by enteroviruses (EVs) of the family Picornaviridae, and has a prevalence pattern typical of enteroviral disease, with peaks in the summer and fall. Enterovirus 71 (EV71), coxsackievirus A16 (CA16), and coxsackievirus A6 (CA6) are the major causative agents of HFMD [91].

The infection is characterized by several days of fever and vomiting, ulcerative lesions of the buccal mucosa, tongue, palate, and gums, and lesions of the hands and feet, which are usually vesicular and occur on the dorsal surfaces, but they may also occur on the palms and soles [92].

Although the initial viral illness is self-limited, it is sometimes followed by AM, meningoencephalitis, or even an acute flaccid paralysis. The reported numbers of meningitis caused by CVA6 and CVA16 have been far fewer than those caused by EV71 [91]. In fact, AM is also a main neurological presentation in EV71 infection. AM from EV71 is clinically indistinguishable from that of other viruses that present with headache, fever, and recurrent emesis without any other neurological manifestations. Although EV71 AM is usually considered a more benign condition compared with brainstem encephalitis, patients with clinical AM from EV71 infection may show brainstem lesions on MRI [92].

## 4. Case Report 1

A 34-year-old Caucasian woman, unemployed, with a medical history of Graves-Basedow disease was treated in our Oral Medicine Unit, “Federico II” University of Naples, Italy, because of severe mucocutaneous PV, with conventional immunosuppressive therapy made up of prednisone (100 mg PO qd) and azathioprine (150 mg PO qd), entering into complete clinical remission. Throughout the tapering of the conventional therapy, after 6 months, she relapsed with the onset of bullous widespread lesions of the oral mucosa and an erosive lesion of the navel. Due to unresponsiveness to conventional therapy and the presence of iatrogenic Cushing syndrome, the patient received IVIg therapy at a dosage of 2 g/Kg/cycle in a standard regimen.

During the first day of the first cycle, after the administration of 50 mL of solution, she developed severe headache, nausea, vomiting, drowsiness, and laryngospasm. Kerning’s and Brudzinski’s signs were negative. The infusion was stopped and the symptoms disappeared. After one hour, the infusion was restarted and the rate of infusion was reduced from 175 to 75 mL/h. All the symptoms reappeared within 30 min; thus, the infusion was stopped again, and the patient was discharged home on therapy (acetaminophen, 1000 mg PO tid and acetylsalicylic acid, 500 mg PO bid), obtaining a complete resolution of symptomatology after two weeks.

Three weeks later, the patient started a new cycle and received IVIg with a modified premedication, adding clordemetildiazepam (2 mg IV) and salicylic acid (500 mg tablets). Additionally, the infusion speed was reduced to 75 mL/hour from the beginning, and the cycle was scheduled over five days. After the administration of 100 mL of solution, the patient developed severe headache, persistent nausea, and vomiting. Kerning’s and Brudzinski’s signs were negative. The infusion was interrupted, and the patient was discharged home with the previous described therapy.

One month later, the patient started a new cycle and IVIg was administered, diluted with saline (1:0.5). After the administration of 300 mL of diluted solution, she developed severe headache with nuchal rigidity, drowsiness, severe photophobia, painful eye movement, nausea, vomiting, and cutaneous rush. Kerning’s and Brudzinski’s signs were positive.

Since the oral medicine specialized team, who was treating the patient, suspected a DIAM, a neurological consultation was requested. Blood tests and MRI of the CNS revealed no acute abnormalities. A lumbar puncture was performed. The cerebrospinal fluid (CSF) was slightly cloudy, with an opening pressure of 210 mm H_2_O. Leukocyte count was 750 mm^3^ (lymphocytes: 11%, neutrophils: 81%, eosinophils: 2%), CSF proteins were 95 mg/dL, CSF glucose was 64 mg/dL, and CSF bacterial, fungal, and viral cultures were negative.

These findings excluded acute bacterial or viral meningitis and confirmed the hypothesis of IVIg-induced AM. The patient definitively ceased the IVIg treatment.

She recovered over the next 48 h and was discharged.

The patient has been followed up to the present time at our unit and she has restarted steroid therapy. The patient gave explicit consent for her case to be published.

## 5. Case Report 2

A 40-year-old Caucasian woman, an otherwise healthy office worker, was followed in our Oral Medicine Unit, “Federico II” University of Naples, Italy, for the diagnosis and treatment of recurrent oral ulcers. After two years of topical steroidal therapy, the patient presented to our observation with a 2-day history of severe headache, drowsiness, malaise, photophobia, phonophobia, nausea, vomiting, and neck stiffness. She appeared to be alert. Vital signs were within normal limits. She had no prior history of headaches. She was taking no drugs at presentation.

Since the oral medicine specialized team suspected a neurological impairment, as the first sign of BD, the patient was referred to the emergency department, where an MRI of the brain and a lumbar puncture were performed. The MRI of the brain was unremarkable.

Cerebrospinal fluid analysis showed elevated protein levels, increased neutrophils, normal opening pressure, and normal glucose levels. Gram stain, bacterial cultures, and PCR for HSV, EBV, and CMV were negative. Furthermore, immunogenetic analysis showed positivity for HLA-B51 allele, and the skin-prick test showed a positive result.

Based on these findings, the patient was diagnosed with acute AM as the presenting feature of NBD.

The patient received high-dose intravenous methylprednisolone (250 mg every 6 h) for 5 days, followed by oral prednisolone (30 mg twice daily). On the third day of treatment, her neurologic symptoms dramatically improved. The patient completely recovered and she was discharged after 1 week. Oral prednisolone was progressively decreased and discontinued one month later. The patient has been followed up to the present time at our unit for BD treatment. The patient gave explicit consent for her case to be published.

## 6. Discussion and Conclusions

Up to the present time, to the best of our knowledge in the scientific literature, this is the first paper aiming to summarize all the potential causes of AM in oral medicine field.

The first case report described a PV patient with recurring and long-lasting IVIg-induced AM, which required the patient to cease the completion of her treatment cycle three times, despite varying the infusion rate, spreading the treatment over more days, and changing the premedication protocol [93]. Obviously, in the present case, the aseptic meningitis was related to the treatment with IVIg, and not to the systemic disease.

Our observation poses the question whether IVIg-induced AM might be the major contraindication to IVIg treatment, and thus prevent the therapy, unlike other serious side effects, such as thromboembolic events, which allow the therapy to be re-started.

Up until now, this is the second case of IVIg-induced AM in a PV patient. In fact, only Ventura et al. published a case of a 26-year-old woman with a 5-year history of a severe and recalcitrant pemphigus vulgaris, treated with IVIg (2 g/kg over three days) in combination with prednisolone (40 mg/day). On the third day of IVIg, she presented with severe headache, nuchal rigidity, photophobia, and nausea. CSF analysis showed a raised white cell lymphocyte count (80/µL) with normal glucose and protein contents. CSF culture and polymerase chain reaction for common viruses were both negative. After infusion discontinuation, she recovered without any medication and was discharged after four days [46].

The second case report described a patient with a medical history of recurrent oral ulcers, in which a sudden onset of AM allow us to diagnose NBD.

Neurological impairment in some cases presents at the moment of the first appearance of the systemic BD; infrequently, it comes first; but usually, it arises throughout the course of the recognized disease [21].

Since AM may be the first presenting symptom of BD, it could be extremely difficult to identify this disorder initially. In the present case, fortunately, it occurred in a patient with a prior medical history of recurrent oral ulcers, which helped us to diagnose the disorder as NBD.

Therefore, when viral infection has already been excluded, BD must always be considered in a differential diagnosis of acute meningeal syndrome, particularly in the presence of systemic signs, such as skin and mucosal lesions, malaise, fatigue, and arthralgia [21].

Finally, BD is typically treated acutely with high-dose intravenous corticosteroids followed by oral prednisone [14]. Our patient showed an excellent response to corticosteroid therapy.

As it is possible to observe from the analysis of the reported cases and from the study of the current literature on the matter, in oral medicine, AM is a rare condition and can pose a diagnostic and management dilemma.

In Table 3 we selected cases where oral involvement was present prior to the onset of AM or at presentation of the disease.

In detail, among eight patients affected by AM–NBD-related, three presented RAS both prior the onset of AM and at AM presentation, three showed RAS only prior the onset of AM, and two displayed RAS at AM presentation. In addition, among eight patients suffering from AM-SS-related, two presented with trigeminal neuropathy, both prior the onset of AM and at AM presentation, one presented with SICCA syndrome prior the onset of AM and with lymphocytic infiltration pattern at minor salivary glands biopsy at AM presentation, four showed lymphocytic infiltration patterns at minor salivary glands biopsy at AM presentation and one presented with SICCA syndrome prior the onset of AM.

Increasing awareness of this possible correlation can help physicians to make a prompt diagnosis, allowing a more effective clinical management and an early detection of complications. The role of the oral medicine specialist in this context is crucial for the following issues: (a) the oral mucosa can represent the first sign of the onset of the systemic disease, (b) the mouth can be involved during the course of the systemic disease and can underline the quality of life of patients, (c) when a suspected drug is present and infections have been ruled out, a DIAM should be taken into consideration.

Therefore, maintaining a high index of circumspection and requesting the appropriate investigation may be extremely useful in unexplained AM.

Since the entities reported in the present review are clinically distinct, it is fundamental to approach a case of AM with a broad differential diagnosis in order that all treatable and preventable causes can be considered.

Discussing these diseases together may provide oral medicine specialists with the key elements to make a challenging diagnosis.

## Figures and Tables

**Table 1:** Main causes of non-infective aseptic meningitis.

Non-Infective Aseptic Meningitis		
Systemic Diseases	Tumors	Drugs
Bechet’s disease *	Solid tumors	Antimicrobials *
Vogt–Koyanagi syndrome	Breast cancer	NSAIDs *
Sarcoidosis *	Small-cell lung cancer	IVIGs *
PTLDs *	Melanoma *	Vaccines
Rheumatoid arthritis *	CUP	Intrathecal medications
Systemic lupus erythematosus *	Gastrointestinal cancer	Monoclonal antibodies *
Vasculitis *	Urinary tract cancer	Ranitidine
Sjögren syndrome *	Hematological tumors *	Carbamazepine *
	B-cell lymphoma	Lamotrigine *
	Acute lymphocytic leukemia	Azathioprine *
	Primary CNS tumors	Allopurinol
	Ependymomas	Sulfasalazine *
	Medulloblastomas	
	Primary CNS lymphomas	

Legends: PTLDs: post-transplantation lymphoproliferative disorder; CUP: carcinoma of unknown origin; CNS: central nervous system; NSAIDs: nonsteroidal anti-inflammatory drugs; IVIGs: intravenous immunoglobulins. * Conditions/drugs potentially oral medicine related.

**Table 2:** Aseptic meningitis: review of literature.

Authors/Year	Number of Cases of AM	Total Sample
**Neuro-Behçet’s Disease**		
Akman-Demir et al., 1999 [16]	1	200
Kidd et al., 1999 [17]	4	50
Mullins et al., 2009 [11]	1	CR
Kikuchi et al., 2010 [18]	1	CR
Gritti et al., 2011 [10]	1	CR
Dutra et al., 2012 [13]	6	36
Starr and Smith, 2017 [19]	1	CR
Watanabe et al., 2018 [20]	1	CR
Kara et al., 2006 [21]	1	CR
Toledo-Samaniego et al., 2020 [22]	11	57
Sakakibara et al., 2009 [23]	1	CR
Alkan et al., 2019 [14]	1	CR
Bir et al., 2005 [24]	1	LE
**Sjögren syndrome**		
Alexander and Alexander, 1983 [25]	5	Data not available
Rossi and Saddi, 2006 [26]	1	CR
Basheer et al., 2019 [27]	1	CR
Akiyama et al., 2020 [28]	2	CR
Mauch et al., 1993 [29]	1	20
Ozasa et al., 2020 [30]	1	CR
Zhao et al., 2020 [31]	1	CR
Alfaro-Giner et al., 1992 [32]	1	CR
Ishida et al., 2007 [33]	1	LE
Moreira et al., 2015 [34]	1	93
**Intravenous Immunoglobulin**		
Kato et al., 1988 [35]	1	LE 1 pt with immune thrombocytopenia
Sekul et al., 1994 [36]	6	54 pts with various immune-related neuromuscular diseases
Rao et al., 1992 [37]	1	LE 1 pt with immune thrombocytopenia
Obando et al., 2002 [38]	2	CR 2 pts with immune thrombocytopenia
Ellis et al., 1994 [39]	1	CR 1 pt with myasthenia gravis
Jain et al., 2014 [40]	1	CR pt with Guillain-Barré syndrome
Watson et al., 1991 [41]	2	CR 2 pts with thrombocytopenia
Kemmotsu et al., 2011 [42]	4	384 pts with Kawasaki disease
Meiner et al., 1993 [43]	1	CR 1 pt with myasthenia gravis
Bharath et al., 2015 [44]	8	1324 pts with various conditions 11,907 IVIg infusions
Waheed et al., 2019 [45]	4	438 with neuromuscular disease
Ventura et al., 2013 [46]	1	1 pts with pemphigus vulgaris
Wanigasekera et al., 2017 [47]	1	CR 1 renal transplant pt
Vassalini et al., 2019 [48]	1	CR 1 pt with acute EBV infection and thrombocytopenia
Wright et al., 2008 [1]	1	CR 1 renal transplant pt
Graça et al., 2018 [49]	1	CR 1 pt with Systemic Lupus Erythematosus
Chaabane et al., 2012 [50]	1	LE 1 pt with idiopathic thrombocytopenia
**Viral infections**		
Read and Kurtz, 1999 [51]	25 HSV-1 related	1683 CSF samples
Eisenstein et al., 2004 [52]	1 HSV-1 related	CR
De Ory et al., 2013 [53]	3 HSV-1 related	194 with viral meningitis
Akya et al., 2015 [54]	3 HSV-1 related	196 with suspected meningitis
Benjamin et al., 2013 [55]	2 HSV-1 related	53 CSF samples
Moon et al., 2014 [56]	8 HSV-1 related	70 pts with aseptic meningitis
Nahdi et al., 2012 [57]	3 HSV-1 related	58 CSF samples
Chang et al., 2007 [58]	2 HFMD related	17 HFMD pts
Liu et al., 2000 [59]	3 EV71 related	119 EV71 infections
Yang et al., 2020 [60]	29 CA6 related	55 pts with severe CA6-associated HFMD
Lo et al., 2011 [61]	1 CA6 related	296 CA6 infection
McMinn et al., 2001 [62]	5 EV71 related	14 cases of EV71-associated neurological disease
Hu et al., 2015 [63]	74 EV71 related	134 cases of EV71-associated neurological disease
Roohandeh et al., 2013 [64]	14 EV71 related	100 CSF samples
Fujimoto et al., 2002 [65]	28 HFMD related	30 HFMD pts
Ho et al., 1999 [66]	5 EV71 related	78 EV71severe infections
Huang et al., 1999 [67]	3 EV71 related	41 EV71 infections
Komatsu et al., 1999 [68]	5 EV71 related	12 cases of EV71-associated neurological disease
Huang et al., 2015 [69]	28 EV71 related 2 CA16 related	1508 HFMD pts

Legends: CR: case report; LE: letter to the editor; pt/pts: patient/patients; IVIg: intravenous immunoglobulin; HSV: herpesvirus; CSF: cerebrospinal fluid; HFMD: hand, foot and mouth disease; EV71: enterovirus 71; CA6: coxsackievirus A6.

**Table 3:** Selected cases of aseptic meningitis and head and neck involvement from literature.

Authors/Year	Etiology	Previous Signs and Symptoms	AM Signs and Symptoms at Presentation	Head and Neck Involvement
Gritti et al., 2011 [10]	Behçet’s disease	RAS, genital ulceration	Fever, headache, pain and stiffness of the neck, impaired vision of the left eye	Headache, neck pain
Kikuchi et al., 2010 [18]	Behçet’s disease	RAS	Headache, fever, RAS	RAS, headache
Mullins et al., 2009 [11]	Behçet’s disease	RAS, recurrent knee synovitis	Headache, confusion, chest pain, fever, drowsiness, RAS, skin rash	RAS, headache
Starr and Smith, 2017 [19]	Behçet’s disease	Data not available	Fever, malaise, photophobia, phonophobia, headache, nausea, vomiting, neck stiffness, RAS, genital ulcers	RAS, headache, neck stiffness
Kara et al., 2006 [21]	Behçet’s disease	Growth retardation, arthralgia, scrotal ulcers, nodular dermal lesions, RAS	Headache, fever, vomiting, RAS, bilateral vitritis, papilledema, retinal hemorrhage	RAS, headache
Sakakibara et al., 2009 [23]	Behçet’s disease	None	Headache, fever, vomiting, neck stiffness, RAS, genital ulcers	RAS, headache
Alkan et al., 2019 [14]	Behçet’s disease	RAS, arthritis	Headache, diplopia, papilledema	Headache
Bir et al., 2005 [24]	Behçet’s disease	RAS, genital ulceration	Headache, papilledema, neck stiffness, venous sinus thrombosis, intracranial hypertension	Headache, intracranial hypertension
Rossi and Saddi, 2006 [26]	Sjögren syndrome	SICCA syndrome	Headache, pain, and stiffness of the neck, diplopia	Lymphocytic infiltration pattern at minor salivary glands biopsy, headache, neck stiffness
Akiyama et al., 2020 [28]	Sjögren syndrome	Case report 1: recurrent AM	Case report 1: fever, headache, sore throat	Case report 1: lymphocytic infiltration pattern at minor salivary glands biopsy, headache
		Case report 2: xerophthalmia	Case report 2: headache, fever, vomiting, polyarthralgia	Case report 2: lymphocytic infiltration pattern at minor salivary glands biopsy, headache
Basheer et al., 2019 [27]	Sjögren syndrome	SICCA syndrome, arthralgia, fatigue	Headache, photophobia, vomiting, neck and shoulder pain	Headache, neck pain.
Ishida et al., 2007 [33]	Sjögren syndrome	Recurrent AM	Headache, nausea, fever, neck stiffness	Lymphocytic infiltration pattern at minor salivary glands biopsy, headache
Ozasa et al., 2020 [30]	Sjögren syndrome and mixed connective tissue disease	Bilateral pain and numbness in the maxillary anterior gingiva and eyelids, generalized body pain	Headache, fever	Trigeminal neuropathy, headache.
Zhao et al., 2020 [31]	Sjögren syndrome	Arthritis, glucosuria	Headache, fever, nausea, vomiting	Lymphocytic infiltration pattern at minor salivary glands biopsy, headache
Alfaro-Giner et al., 1992 [32]	Sjögren syndrome and mixed connective tissue disease	Trigeminal neuropathy	Fever	Facial pain and hypoesthesia, SICCA syndrome
Ventura et al., 2013 [46]	IVIg-induced in pemphigus vulgaris patient	Cutaneous and mucosal bullae	Headache, neck stiffness, photophobia, nausea	Oral mucosal blisters

Legends: RAS: Recurrent aphthous stomatitis.

## Data Availability

Data are contained within the article.
